# Influenza Associated Encephalopathy, Encephalitis, and Acute Necrotizing Encephalitis in Adults: A Scoping Review of 83 Cases

**DOI:** 10.3390/neurolint18070140

**Published:** 2026-07-22

**Authors:** Veljko Rabasovic, Milan Radovanovic, Milan Jovanovic, Vladislav Glusac, Nenad Stojiljkovic, Varun Jain, Natasa Radovanovic, Bojana Milekic, Charles W. Nordstrom, Igor Dumic

**Affiliations:** 1 School of Medicine, University of Belgrade, 11000 Belgrade, Serbia; rabasovicveljko@gmail.com (V.R.); vladaglu@gmail.com (V.G.); 2 Department of Medicine, Dartmouth Hitchcock Medical Center, Dartmouth College, Hanover, NH 03755, USA; radovanovic.milan@mayo.edu (M.R.); natasa.stankovic.md@gmail.com (N.R.); 3 Clinic for Neurology, Klinikum Bremerhaven Reinkenheide gGmbH, 27574 Bremerhaven, Germany; jovmilanovic@gmail.com; 4 Neurology Residency Program, Department of Neurology and Pain Medicine, Immanuel Klinik Rudersdorf, Medical University Brandenburg Theodor Fontane, Rudersdorf bei Berlin, Brandenburg 15562, Germany; 5 Neurology Fellowship Program, Department of Neurology, School of Medicine, University of Louisville, Louisville, KY 40292, USA; nenadstojiljkovic1997@gmail.com; 6 Department of Neurology, Mount Sinai Hospital, New York, NY 10029, USA; varun.jain@mssm.edu; 7 Department of Critical Care Medicine, Mount Sinai Hospital, New York, NY 10029, USA; bojana.milekic@mssm.edu; 8 Department of Hospital Medicine, Mayo Clinic Health System, Eau Claire, WI 54703, USA; nordstrom.cw@mayo.edu

**Keywords:** influenza, encephalopathy, encephalitis, acute necrotizing encephalitis, adults, neuro-infectious disease, cytokines

## Abstract

**Background**: Influenza-associated encephalopathy, encephalitis, and acute necrotizing encephalopathy (ANE) are rare but potentially life-threatening neurological complications of influenza. Adult cases remain poorly characterized because the available literature is largely limited to isolated case reports and small case series. **Methods**: A PRISMA-guided scoping review of the MEDLINE database was conducted through 31 May 2026. Published adult cases of influenza-associated encephalopathy, encephalitis, and ANE were identified and analyzed for demographic characteristics, clinical presentation, neuroimaging findings, treatment, and outcomes. **Results**: Eighty-three adult cases reported between 1958 and 2026 were included. The mean age was 45.6 ± 17.8 years, and 57.8% were male. Two-thirds did not have any underlying comorbidities. Neurological symptoms developed a mean of 4.6 days after influenza onset, with influenza A accounting for 80.7% of infections. Fever (91.6%), altered mental status (86.7%), and seizures (36.1%) were the most common manifestations. Encephalitis was the predominant presentation (44.5%), followed by encephalopathy (31.3%) and ANE (24.1%). MRI most frequently demonstrated cerebral hemispheric lesions (53.0%) and bilateral thalamic involvement (36.4%), while EEG abnormalities were reported in 69.6% of patients. Overall mortality was 22.9%, highest among patients with ANE (45.0%). Among survivors, 28.9% experienced persistent neurological sequelae. **Conclusions**: Influenza-associated encephalopathy, encephalitis, and ANE are rare in adults but are associated with substantial morbidity and mortality. This review represents the largest adult cohort reported to date and provides important insights into the clinical spectrum, neurodiagnostic findings, and outcomes of these uncommon complications. This review highlights significant gaps in knowledge and the need for collaborative multicenter studies to improve the diagnosis, treatment, and outcome of these severe complications of influenza infection.

## 1. Introduction

Influenza is primarily a respiratory pathogen that typically causes mild upper respiratory tract infections during seasonal outbreaks. It is a single-stranded RNA virus belonging to the *Orthomyxoviridae* family. Populations at increased risk for severe infection include children (particularly those under the age of five) and individuals with chronic conditions, such as asthma, hypertension, diabetes mellitus, or those receiving immunosuppressive therapy [1,2]. Pregnant women, residents of long-term care facilities, the elderly, and individuals with medically complicated obesity are also at higher risk for severe disease and complications development [1,2,3,4].

Neurological manifestations associated with influenza are linked to high mortality rates, reaching up to 30% in some studies [3,4]. The most common neurological manifestations include varying degrees of altered mental status, headache, and seizures [1,2,3,4]. More severe influenza-associated neurological syndromes include Reye’s syndrome in both children [1] and adults [5], Guillain-Barré syndrome [6], influenza-associated encephalopathy (IAE) [3], acute necrotizing encephalopathy (ANE) [4], influenza encephalitis, hemorrhagic shock [7,8], and cytokine storm-associated encephalopathy [9,10].

Beyond acute neurological syndromes, influenza is increasingly recognized as being associated with a broad spectrum of psychiatric manifestations, underscoring the complex interactions between viral infection and the central nervous system [11]. During the acute phase of influenza infection, patients may develop delirium, hallucinations (predominantly visual), anxiety, behavioral disturbances, confusion, disorientation, and acute psychosis. Additionally, several observational studies have suggested potential associations between influenza infection and the subsequent development of schizophrenia, mood disorders (including depression), and Parkinson’s disease [11,12,13]. The proposed mechanisms include excessive systemic cytokine responses, blood–brain barrier dysfunction, immune-mediated neuronal injury, and direct viral invasion of the central nervous system. However, the available evidence is largely observational, and a causal relationship has not been established. Collectively, these findings support the concept that influenza-associated encephalopathy and encephalitis exist within a broader spectrum of influenza-related neurological and psychiatric complications rather than as isolated clinical entities [12,13].

Encephalopathy is a broad clinical term describing a syndrome of global brain dysfunction, typically presenting with altered mental status, confusion, or a decreased level of consciousness. It may result from a wide range of causes, including metabolic disturbances (e.g., hepatic or renal failure), hypoxia, exposure to toxins, or systemic infections [14,15,16,17]. Encephalitis, on the other hand, refers specifically to inflammation of the brain parenchyma, most commonly due to infectious (particularly viral), autoimmune, or paraneoplastic etiologies. It is characterized by the presence of encephalopathy accompanied by additional features such as fever, seizures, and focal neurological deficits. In contrast to encephalopathy, where cerebrospinal fluid (CSF) findings are typically normal and there is no CSF pleocytosis, encephalitis is usually associated with abnormal CSF findings, including pleocytosis, and corresponding abnormalities on neuroimaging [14,15,16,17].

Influenza associated encephalopathy, influenza encephalitis, and acute necrotizing encephalitis (ANE) are rare but potentially life-threatening neurological complications of acute influenza infection. Most published studies are retrospective, single-center investigations, case reports, or small case series originating primarily from Asia and in children [2,3,4]. Large multicenter prospective studies are currently lacking, and there is limited research on the pathogenesis and treatment of these conditions, with previous reviews predominantly concentrated on ANE [2,3,4]. The objective of this scoping review is to provide a more comprehensive synthesis of literature by encompassing all reported cases of influenza-associated encephalitis, encephalopathy, and ANE and systematically analyzing available information from case reports and case series. Given the rarity of these neurological complications of influenza, conducting large, prospective studies is unlikely to be practical or feasible. Consequently, despite the inherent limitations of systematic reviews based primarily on case reports and case series, such studies can provide valuable insights into the clinical spectrum, diagnostic approaches, management strategies, and outcomes of these uncommon manifestations. By synthesizing the available evidence, we want to inform future research priorities while also providing clinicians with practical information that may facilitate earlier recognition and improve the management and outcomes of influenza-associated neurological complications.

## 2. Methodology

We performed a scoping review of all influenza-associated encephalopathy, influenza encephalitis, and ANE cases according to preferred reporting items for systematic reviews and meta-analyses (PRISMA) guidelines using the PubMed search engine of the Medline (National Library of Medicine, Bethesda, MD, USA) database, from database inception until 1 May 2026. The systematic review protocol was prospectively registered with the Open Science Framework (OSF) to ensure methodological transparency and reproducibility (DOI: 10.17605/OSF.IO/NPRKD). The quality of the case reports included in the analysis was independently assessed using the Joanna Briggs Institute critical appraisal checklist for case reports, which evaluates methodological rigor and reporting quality across multiple domains.

**Figure 1:**
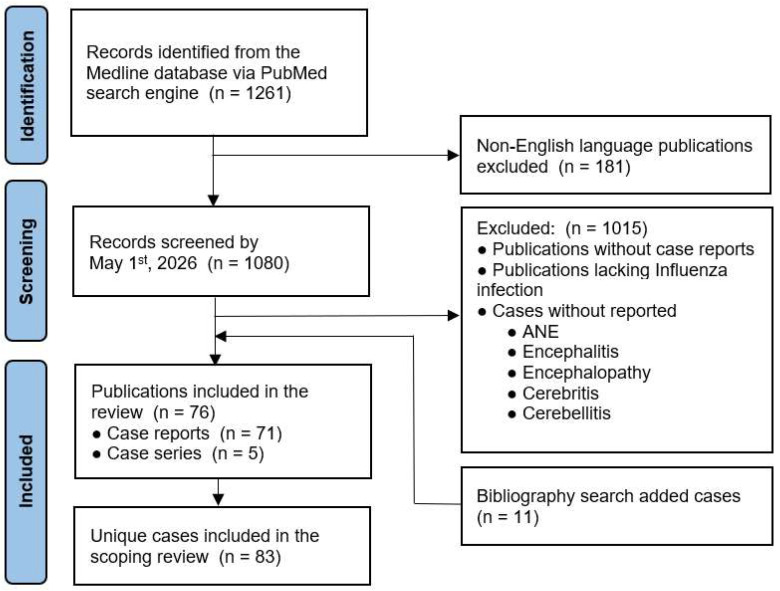
Prisma flowchart detailing the search results.

A total of 1261 original articles were identified that mention the following search keywords (combination of MeSH and non-MeSH terms): “influenza AND encephalitis” OR “influenza AND encephalopathy” OR “influenza AND cerebritis” OR “influenza AND cerebellitis” OR “ acute necrotizing encephalitis AND influenza”. We excluded cases where the diagnosis was uncertain, either because influenza was not definitely diagnosed or there was a lack of evidence of encephalitis and/or encephalopathy. Duplicate articles, articles in languages other than English, abstracts without comprehensive case descriptions, and narrative reviews were all excluded. Two authors (M.R. and N.R.) independently and blindly identified and selected titles, abstracts, and full texts in the database search. Discrepancies in the selected articles were resolved by the third author (I.D.). Additionally, the reference list of selected articles was searched to identify any additional cases for inclusion in accordance with previously established selection criteria. The flowchart of detailed article selection and the final cases included in the analysis is illustrated in Figure 1. We extracted demographic data, comorbidities, presenting symptoms, onset of neurological symptoms relative to influenza diagnosis, physical exam findings, level of consciousness, occurrence of seizures, laboratory studies, imaging findings, treatment options, complications, and outcomes (V.G. and V.R.). Data were analyzed by descriptive statistics and expressed as mean ± standard deviation for continuous data, or as frequency and percentages for categorical data.

## 3. Results

Our scoping review identified 83 unique adult cases from 71 case reports [4,5,8,18,19,20,21,22,23,24,25,26,27,28,29,30,31,32,33,34,35,36,37,38,39,40,41,42,43,44,45,46,47,48,49,50,51,52,53,54,55,56,57,58,59,60,61,62,63,64,65,66,67,68,69,70,71,72,73,74,75,76,77,78,79,80,81,82,83,84,85] and 5 case series (each describing 2–3 patients) [86,87,88,89,90], published between 1958 and 2026.

**Figure 2:**
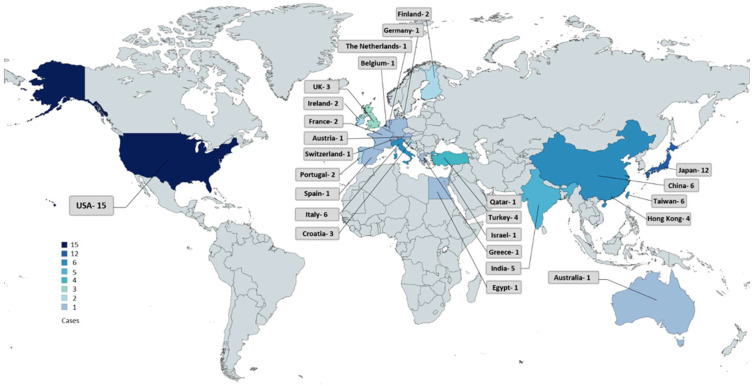
Geographic distribution of reported adult influenza-associated encephalopathy and encephalitis cases included in the review, by country and territory.

These cases represent a diverse international cohort (Figure 2). Out of the 83 patients, 48 (57.8%) were male and 35 (42.2%) female, with a mean age of 45.6 ± 17.8 years. Most patients (65.1%) had no comorbidities, and the mean onset of neurological symptoms following viral infection was 4.6 days. Neurological severity was assessed using the Modified Rankin Scale (MRS), with a mean score of 3.4 at admission, improving to 2.3 at discharge. The most common clinical feature was fever, reported in 76 cases (91.6%), followed by altered mental status in 72 cases (86.7%), including lethargy in 24 (28.9%), stupor in 16 (19.3%), and coma in 24 (28.9%). Convulsive seizures occurred in 30 cases (36.1%), headache in 22 cases (26.5%), motor deficits in 20 (24.1%), cranial nerve involvement in 15 (18.1%), shock and dysarthria in 12 (14.4%), aphasia in 11 (13.2%), and sensory abnormalities in 8 cases (9.6%). Among the 83 analyzed cases, encephalitis was the most common neurological presentation, occurring in 37 patients (44.5%), followed by encephalopathy in 26 patients (31.3%), while ANE was identified in 20 patients (24.1%). Mortality was highest among patients with ANE (45%), followed by encephalopathy (19.2%) and encephalitis (14.8%).

Magnetic resonance imaging (MRI) results were available for 66 cases: cerebral hemispheres were involved in 35 cases (53%), with bilateral thalamic lesions seen in 24 cases (36.4%). Electroencephalography (EEG) was performed in 56 cases, revealing abnormal findings in 39 (69.6%).

Influenza infection was identified by molecular testing of nasopharyngeal swab or CSF specimens. However, the exact molecular assay used was inconsistently reported in the analyzed case reports. Influenza virus type was reported in 78 cases, with 63 (80.7%) infected with influenza A, 14 (17.9%) with influenza B, and one case (1.3%) co-infected with both. Out of 49 cases with strain-level data, 35 (71.4%) were infected with H1N1, 13 (26.5%) with H3N2, and 1 case of H3 (2%). Laboratory findings, including IL-6, were reported in 12 cases, and were elevated in 10 (83.3%). Influenza virus was isolated from the CSF in 7 out of 83 cases, corresponding to 8.4% of the reviewed cases. Treatment included oseltamivir in 61 cases (73.5%), while peramivir was used in three cases; the remaining patients received no antiviral treatment. Steroids were given in 29 cases (34.9%), a combination of steroids and intravenous immunoglobulin (IVIG) in 17 (20.5%), steroids plus plasmapheresis in 5 (6%), IVIG alone in 1 case (1.2%), and triple therapy (steroids, IVIG, and plasmapheresis) in 1 case (1.2%). Hospitalization duration was reported in 59 cases, with a median stay of 28 days (range 2–150 days). Clinical outcomes were available for all 83 cases: 24 patients (28.9%) recovered with neurological sequelae, 19 (22.9%) died during the acute phase, and the remaining cases recovered without sequelae.

## 4. Discussion

In this scoping review of adults with IAE, influenza encephalitis, and ANE, we identified 83 patients who had definitive influenza diagnosis and exhibited neurological complications. Unlike for other severe neurological complications, most patients (65.1%) had no underlying comorbidities, and the mean interval between influenza infection and onset of neurological symptoms was 4.6 days, with a mean hospitalization length of 28 days. Seizures were reported in 36.1%, while 18.1% of patients presented with shock. Neuroimaging demonstrated abnormalities, with MRI most frequently showing cerebral hemispheric and bilateral thalamic lesions. Electroencephalography, performed in 56 patients, showed abnormal findings in 69.6%. Influenza A accounted for 80.7% of infections, with H1N1 identified in 71.4% of cases. Elevated interleukin-6 (IL-6) levels were observed in 10 out of 12 reported cases. Influenza virus detection in CSF was uncommon, occurring in only 8.4% of cases. Most patients received antiviral therapy in combination with various immunosuppressive treatments. Overall mortality was 22.9% (19 patients).

Adult-focused studies on influenza-associated encephalopathy, encephalitis, and ANE remain limited despite the extensive literature available in pediatric populations. A summary of the key adult studies is presented in Table 1 [4,41,91,92,93].

Pathogenesis of influenza-associated neurological complications remains unclear. Although various pathogens and vaccines can result in ANE, the clinical manifestations of ANE do not differ significantly in relation to etiology [94]. Some proposed mechanisms include cytokine storm, genetic susceptibility, dependent enhancement of vaccine-related antibodies, direct pathogen invasion, and blood–brain barrier (BBB) dysfunction. The most prominent of these is thought to be the cytokine storm [94,95]. Cytokines that might be elevated in IAE, influenza encephalitis, and ANE include tumor necrosis factor-alpha (TNF-α), interleukin-1 beta, IL-2, IL-6, and IL-15. Those with the dominant effect include IL-6 and TNF-α [96,97]. Elevated cytokine levels can cause an imbalance in glutamatergic and GABAergic neurotransmission and lead to nervous system damage due to excitatory toxicity [98]. IL-6 has been shown to be neurotoxic and is considered a prognostic factor, even a potential target for ANE therapeutic agents, with several studies conducted on patients who responded well to an IL-6 blocker (tocilizumab) [99,100]. Additionally, elevated TNF-α levels can damage vascular endothelial cells, disrupt the BBB, and induce oligodendroglial necrosis [101,102]. Peripheral inflammation triggered by the aforementioned host defenses is suspected to cause BBB disruption and local inflammatory response in the central nervous system (CNS), which can eventually lead to encephalopathy [103,104,105,106,107,108]. Despite strong support for this hypothesis, the number of cases reporting these cytokines remains low, underscoring the need to educate clinicians on the importance of these biomarkers and the value of routinely assessing them in relevant clinical settings.

While some viruses cause disease via direct CNS invasion (e.g., viral meningitis), this mechanism remains controversial in influenza encephalitis and ANE. Notably, influenza virus is rarely isolated from CNS tissues or cerebrospinal fluid (CSF) [109], although one Japanese study detected influenza RNA in the CSF of most patients [110]. Given the infrequent detection of influenza in CSF, the mechanism by which influenza may invade or affect the CNS remains unclear.

Infectious encephalitis is most commonly viral in origin, with herpesviruses, arboviruses, enteroviruses, and adenoviruses accounting for the majority of cases worldwide. Herpes simplex virus (HSV) predominates among older adults, whereas Japanese encephalitis virus remains the leading cause in many Asian regions. Bacterial, fungal, and parasitic etiologies are relatively rare [14]. Influenza A has been associated with ANE and ADEM, the latter potentially being post-vaccinal. ADEM typically presents with large, asymmetric, multifocal inflammatory demyelination affecting the white matter of the brain and spinal cord, whereas ANE is characterized by bilateral, symmetrical lesions, mainly in the thalami [64].

Our findings are consistent with those reported by Chen who reviewed 22 cases of influenza-associated ANE [4]. More than 70% cases in both studies were infected by influenza A, and a median interval of 3 days between the onset of prodromal symptoms and the development of neurological symptoms in Chen’s study is somewhat shorter that 4.6 days found in this review. The pattern of neuroimaging is also comparable between the two studies. Bilateral thalamic involvement was the most frequent finding, followed by lesions in the cerebral hemispheres, brainstem, cerebellum, basal ganglia, and corpus callosum. Various treatment options were used in both studies, most commonly corticosteroids alone or in combination with intravenous immunoglobulin (IVIG). Regardless of the treatment option, the mortality rate was 30%, which is comparable with our findings [4].

A pediatric study in Italy examined 25 children with influenza-associated IAE, with influenza A being the most common pathogen consistent with prior studies [111]. Unlike our adult cases, this study reported a 100% survival rate and only one severe complication. Neuroimaging was carried out in 14 patients, but abnormalities appeared in only two, reflecting generally mild to moderate disease in the pediatric population. In contrast, in our systematic review of adult cases, neuroimaging was conducted more frequently and was often abnormal, which might be associated with the higher mortality described in this cohort compared to pediatric cases.

Management of patients with influenza-associated neurological complications has not been evaluated in clinical trials, and recommendations are based on expert opinion, case series, and retrospective cohort study data. High-dose intravenous (IV) corticosteroids are the cornerstone of ANE therapy. Most series report using pulse methylprednisolone (commonly 20–30 mg/kg/day for 3–5 days) initiated as early as possible (often within 24 h of neurologic decline) [112,113]. Early steroid administration correlates with significantly better outcomes [111]. Chang et al. found that 87.5% of the children without brain stem lesions recovered well if treated within 24 h. In many cases, steroids are tapered over weeks (e.g., by oral prednisolone), but the optimal duration is not standardized [112].

Intravenous immunoglobulins (IVIGs) are often administered (usually 2 g/kg over 2–5 days) as adjunctive therapy in pediatric ANE. For example, a multicenter series from Saudi Arabia found 83% of children received combined IV steroids and IVIGs, although this study did not comment on the efficacy of this treatment [114]. However, recent meta-analysis showed no significant improvement in outcomes with IVIG use, and combining IVIGs with early steroids did not add benefit over steroids alone [113]. Other immunotherapies (e.g., tacrolimus) have been tried in isolated reports but lack evidence of efficacy.

Plasmapheresis (PLEX) is used in severe or refractory ANE cases, often when patients fail to improve on steroids. Some reports suggest that PLEX may improve survival: in one pooled analysis, all 18 patients who received PLEX survived, versus 45% survival without PLEX [113]. Still, PLEX is rarely reported (typically reserved for critically ill children) and lacks controlled evidence. Some case reports argue for the earlier initiation of PLEX as a strategy for better outcomes while commenting on the fact that this may be difficult in many hospitals where the early initiation of PLEX could take an extended amount of time due to multi-specialty approach requirements [113,114,115].

Given ANE’s cytokine-storm pathophysiology, several reports describe anti-cytokine agents with some success [116]. IL-6 receptor antagonist (tocilizumab) has been administered in some severe ANE cases [116,117]. The use of IL-1 blockade (anakinra) has also been reported in isolated cases, primarily somewhat inspired by its use in febrile infection-related epilepsy syndrome (FIRES) with positive outcomes [118].

In the reported literature, all cytokine-blockade strategies are experimental: dosing regimens are not standardized, and the risk of infection (due to immunosuppression) is a concern. No formal guidelines exist, so such therapies are used at the clinicians’ discretion in refractory cases. Because ANE follows viral prodromes (influenza, HHV-6, SARS-CoV-2, etc.), empirical antimicrobials are given early. Influenza-associated ANE patients typically receive neuraminidase inhibitors (oseltamivir or peramivir) promptly, though no trial has proven efficacy. Supportive management (seizure control, mechanical ventilation, hemodynamic support) is often required. The most severe neurological complications like cerebellar edema and elevated intracranial pressure are managed with osmotherapy, deep sedation/analgesia, hyperventilation, or normothermia. Overall, supportive ICU care for the most complex cases is essential [119]. Those cases mostly require prolonged mechanical ventilation, tracheostomy, and management of multi-organ failure with prolonged hospital stay and recovery.

There are currently no international evidence-based guidelines for ANE. Published recommendations are largely expert opinions [112].

The optimal management of ANE has yet to be established. No randomized trials have defined optimal therapy, so treatment is based on small case series and expert opinion. Finally, pediatric versus adult ANE may differ; adult reports (often caused by influenza or COVID-19) use similar immunotherapies, but there are limited data on age-specific protocols or outcomes [4].

## 5. Limitations

This scoping review has several limitations. First, the available evidence is derived predominantly from case reports and small case series, which are inherently susceptible to reporting bias and have limited generalizability. In addition, substantial heterogeneity exists across the published literature with respect to diagnostic criteria, clinical definitions, and reported outcomes. Furthermore, key clinical variables, including cerebrospinal fluid findings and inflammatory biomarkers such as interleukin-6 (IL-6), were inconsistently reported, precluding robust causal inference and limiting the feasibility of multivariable regression analyses.

Second, there is a significant risk of publication bias, as more severe or atypical cases are more likely to be published, which may have contributed to an overestimation of mortality and morbidity. In addition, the review is limited to studies published in English, introducing potential language bias and the exclusion of relevant data from non-English literature.

Third, most cases were retrospectively described, limiting the ability to infer causality or accurately assess incidence and risk factors. The rarity of influenza detection in cerebrospinal fluid and the selective availability of advanced immunological testing further restrict mechanistic interpretations.

Finally, variability in reporting standards and incomplete clinical data across studies limit the ability to perform robust quantitative synthesis. These limitations highlight the need for standardized reporting and prospective, multicenter studies to better define the spectrum and pathophysiology of influenza-associated neurological complications.

## 6. Conclusions

In this scoping review of 83 patients with severe neurological complications from influenza, two-thirds did not have comorbidities, and overall mortality was 23%. The mean hospital length of stay was 28 days, underscoring substantial healthcare utilization, and nearly 30% of survivors were discharged with persistent neurological sequelae, indicating a significant long-term burden. Although CSF inflammatory markers were inconsistently reported, most patients in whom IL-6 was measured demonstrated elevated levels, while influenza virus was rarely isolated from CSF. These findings suggest that disease severity may not correlate with direct viral invasion or overt CSF inflammation, supporting a predominantly cytokine-mediated pathophysiological mechanism.

The relatively high mortality observed may partly reflect selection bias toward more severe published cases. Considerable heterogeneity in clinical presentation, diagnostic workup, and management underscores persistent gaps in evidence. Multicenter prospective studies are needed to better elucidate disease mechanisms, standardize diagnostic approaches, identify prognostic biomarkers, and develop targeted therapeutic strategies for these severe neurological complications.

## Figures and Tables

**Table 1:** Summary of adult studies on influenza-associated encephalopathy and encephalitis.

Year	Reference	Type of Study	Cohort Size	Main Finding	Mortality
2024	4	Literature review	22	Bilateral thalamic lesions were the most common MRI finding in adult influenza-associated ANE.	32%
2016	41	Literature review	44	Adult influenza-associated encephalopathy commonly presented with confusion/seizures and MRI abnormalities.	18%
2025	91	Retrospective observational study	9	Encephalitis was the most common complication.	0%
2019	92	Retrospective nationwide survey	44	Altered mental status occurred in 93% of cases, with fever as the most common initial symptom. Good recovery was achieved in 63% of patients. MRI abnormalities were detected in 45.2% and EEG abnormalities in 86.4% of patients. Hyperglycemia was associated with poor prognosis.	7%
2020	93	Retrospective cohort study	24	Influenza-associated encephalopathy was the most common neurological complication; 96% of cases were associated with influenza B, and most patients recovered completely.	0%

## Data Availability

The raw data supporting the conclusions of this article will be made available by the authors on request.
